# Daratumumab-EPOCH for transformed anaplastic multiple myeloma

**DOI:** 10.1016/j.htct.2025.103977

**Published:** 2025-09-06

**Authors:** Espen Stormorken, Anders Erik Astrup Dahm

**Affiliations:** a Department of Haematology, Akershus University Hospital, Lørenskog, Norway; b Institute of Clinical Medicine, University of Oslo, Oslo, Norway

## Introduction

Anaplastic multiple myeloma is not an established diagnostic entity but is described many times and has distinct histopathological features. It is associated with poor prognosis, high Ki67, rapid growth, and extramedullary myelomas. It can debut as the first symptom of multiple myeloma (primary anaplastic myeloma) or transform from an existing myeloma. Anaplastic transformation of multiple myeloma is typically associated with a survival of only a few months [1,2]. Myeloma treatments are mainly based on dexamethasone, proteasome inhibitors, immunomodulatory drugs, and CD 38 antibodies [3]. Traditional cytotoxic drugs are nowadays rarely used, except melphalan, mostly together with autologous stem-cell transplantation (ASCT). There have been reported cases where primary anaplastic multiple myeloma has responded well to the EPOCH regimen used for lymphoma [4]. There are, however, no reports of successful treatment of transformed anaplastic multiple myeloma. Here we present two heavily treated myeloma patients who had an anaplastic transformation of their disease. They received a combination of modern myeloma treatment with traditional lymphoma treatment with surprisingly good results.

## Case report

Case 1 involved a 64-year-old woman diagnosed in 2015 with multiple myeloma, characterized by an IgG monoclonal protein (Table 1). She had been diagnosed with a monoclonal gammopathy of undetermined significance four years earlier. Her past medical history included rheumatoid arthritis, hypertension, ovarian cancer, herpes zoster, and sclerotic aortic and mitral valves. First line treatment was induction therapy followed by high-dose melphalan with ASCT. Three years later she had a biochemical recurrence. Second-line treatment was a second induction and high-dose melphalan with ASCT followed by maintenance therapy with lenalidomide and dexamethasone (Rd - Table 1). In December 2021 she was diagnosed with transformed anaplastic multiple myeloma (Table 2). She experienced persistent high fever with deteriorating general condition. She received four cycles of daratumumab, carfilzomib, and dexamethasone with a reduction of plasma cells in the bone marrow from 70 % to 40 %, but the fever remained. Four months later, a biopsy from an axillary lymph node confirmed anaplastic multiple myeloma. The EPOCH chemotherapy regimen traditionally used for aggressive lymphomas had been tried previously for anaplastic myeloma [4,5]. We therefore decided to give her daratumumab together with EPOCH, i.e., continuous infusion of etoposide 100 mg/m^2^ Day 1–4, prednisolone 60 mg/m^2^ Day 1–5, vincristine 0.8 mg/m^2^ on Day 1–4, doxorubicin 20 mg/m^2^ Day 1–5, and cyclophosphamide 750 mg/m^2^ Day 5 (Dara-EPOCH). Daratumumab was administered weekly, and EPOCH was administered every third week. She received five cycles of Dara-EPOCH, the last cycle in July 2022. In November 2022 there were 0.5 % plasma cells in the bone marrow and the monoclonal component was zero. A positron emission tomography-computed tomography (PET-CT) scan identified residual lesions in the left iliac bone and a left rib. Radiotherapy for these lesions was initiated in November 2022. At the same time, maintenance treatment was started with daratumumab weekly. That winter she sent us a picture where she went cross-country skiing.

**Table 1:** Baseline patient and disease features at multiple myeloma diagnosis.

	Case 1	Case 2
Sex	Female	Female
Age at multiple myeloma diagnosis	64 years	61 years
Characteristics of the myeloma	Monoclonal IgG protein 41 g/L, 30 % plasma cells in the bone marrow. Anaemia, hypercalcaemia, and skeletal involvement.	Non-secretory myeloma, 90 % plasma cells, hypercalcaemia, renal failure and osteolytic skeletal lesions.
ISS	III	II
Cytogenetics	Small extra marker chromosome	t(11;14)(q13;q32)
1st line treatment	VCd x 4, HDM with ASCT	VCd x 2, VTd x 2, VRd x 2, HDM with ASCT
2nd line treatment	VRd x 4 + HDM with ASCT + 27 x Rd maintenance therapy.	RT, Vd x 1, PomVD x 5, HDM with ASCT, 7 x DaraVd maintenance therapy

VCd: bortezomib, cyclophosphamide, dexamethasone; VRd: bortezomib, lenalidomide, dexamethasone; VTd: bortezomib, thalidomide, dexamethasone; HDM: high-dose melphalan; RT: radiotherapy; PomVd: pomalidomide, bortezomib, dexamethasone; DaraVd: daratumumab, bortezomib, dexamethasone.

**Table 2:** Treatment given after anaplastic transformation and survival.

	Case 1	Case 2
Characteristics of anaplastic transformation	70 % plasma cells in the bone marrow, monoclonal protein IgG of 19 g/L, Ki67 of 100 %, the plasma cells positive for c-MYC protein.	Extramedullary disease, Ki67>60 %, C-Myc protein positive and cyclin D1 positive. Normal bone marrow biopsy.
Age at anaplastic transformation	70 years	66 years
Cytogenetics	Loss of 17p13	Normal
1st line treatment after anaplastic transformation	Dara-Kd	Dara-EPOCH
2nd line treatment after anaplastic transformation	Dara-EPOCH	Daratumumab maintenance therapy
3rd line treatment after anaplastic transformation	Daratumumab maintenance therapy	Dara-modified POMP
4th line treatment after anaplastic transformation	Dara-modified POMP	Belantamab mafodotin
5th line treatment after anaplastic transformation	Belantamab mafodotin	Talquetamab
6th line treatment after anaplastic transformation	Carfilzomib EPOCH	Isa-Kd-IME
Survival after anaplastic transformation	19 months	26 months
Overall survival after multiple myeloma diagnosis	7 years 9 months	8 years

Dara-Kd: daratumumab, carfilzomib, dexamethasone; Dara-EPOCH: daratumumab, etoposide, prednisolone, vincristine, cyclophosphamide, doxorubicin; Dara-modified POMP: daratumumab, dexamethasone, vinblastine, mercaptopurine, methotrexate; Isa-Kd-IME: isatuximab, carfilzomib, dexamethasone, ifosfamide, methotrexate, etoposide.

In January 2023 the monoclonal protein increased and we started treatment based on the POMP regimen [6] with vinblastine every fourth week, dexamethasone Day 1–5 of each 28 day cycle, together with daily oral mercaptopurine and weekly oral methotrexate in addition to the weekly daratumumab with dexamethasone she was already receiving (Dara-modified POMP). In May 2023 she had increased skeletal lesions and was administered two doses of the antibody-drug conjugate belantamab mafodotin. A lesion at the right orbit was treated with radiotherapy in July 2023. The disease continued to progress evidenced by extramedullary lesions and following discussion with the patient we decided to give a new EPOCH cycle with carfilzomib. She died due to febrile neutropenia in late July 2023.

Case 2 was a 61-year-old woman diagnosed with non-secretory multiple myeloma in 2016. She received induction treatment and high-dose melphalan with ASCT (Table 1). She started Rd as maintenance treatment, but developed a rash and the treatment was discontinued. Five years later she was admitted to hospital with malignant spinal cord compression. She was treated with radiotherapy followed by subsequent induction and a second high-dose melphalan with ASCT. A response assessment PET-CT showed a lesion in the left femur. She then received seven cycles of daratumumab-bortezomib-dexamethasone (Table 1). Six months later, in March 2022, the disease recurred with tumours in the left pleura, behind the aorta, and a skeletal lesion in L1. A biopsy showed anaplastic multiple myeloma (Table 2). She received two cycles of EPOCH, and four cycles of Dara-EPOCH. A PET-CT in October 2022, six months after the first EPOCH cycle, demonstrated almost complete remission with a residual lesion in the left femur. She then started weekly daratumumab and the residual lesion in the left femur was treated with radiotherapy.

In January 2023 the treatment was shifted to Dara-modified POMPIn May 2023, a PET-CT revealed progressive disease with bilateral rib involvement, multifocal lesions in all four extremities, and paravertebral tumors. Treatment was changed to belantamab mafodotin which was discontinued after two doses due to keratopathy. In August, the treatment was changed to talquetamab, a bispecific antibody that induces apoptosis of myeloma cells by means of T-cell recruitment and activation [7]. She received 40 mg subcutaneous every 14th day, and the disease stabilized until November 2023 when she was readmitted to the hospital with increasing pain. A PET-CT showed increased size of the myeloma lesions. Treatment was shifted to a 28-day cycle of isatuximab Day 1 and 15, carfilzomib Day 1, 8 and 15, ifosfamide 1000 mg/m^2^ Day 1–5, etoposide (Etoposide) 100 mg/m^2^ Day 1–3, methotrexate (Methotrexate) 30 mg/m^2^ Day 3 with dexamethasone and mesna (Mesna) resulting in effective pain control. A PET-CT in February 2024 showed regression of the myeloma lesions. In April 2024 the chemotherapy no longer provided pain relief. A subsequent PET-CT showed recurrence of the lesions from December 2023. She was transitioned to palliative care and died in May 2024.

## Discussion

These two case reports illustrate that conventional chemotherapy used for aggressive lymphomas combined with anti-myeloma treatment is efficient in the treatment of transformed anaplastic multiple myeloma. The expected survival of patients with transformed anaplastic myeloma is only a few months. In contrast, these two patients lived for 19 and 26 months with a reasonably good quality of life. Most published cases of anaplastic myeloma are from patients who debut with anaplastic myeloma [1,4,8]. Although transformed anaplastic myeloma is a well-known aggressive end stage of myeloma,^2^ we only found one publication reporting on the treatment of two such cases [1]. One of the two cases received thalidomide, vincristine, doxorubicin, dexamethasone (Thal-VAD) and lived for three months. The other received one cycle of bortezomib, cisplatin, cyclophosphamide, etoposide, dexamethasone followed by five cycles of Thal-VAD with at least nine months survival. Previous studies have shown good outcomes with EPOCH (without daratumumab) in patients with anaplastic myeloma at diagnosis, thus guiding its application in our two cases [4,9]. Others have tried more standard myeloma treatment with and without effect [8,10].

A possible reason for the effect of the EPOCH regimen might be that anaplastic myeloma, in contrast to regular myeloma, is a rapidly growing malignancy. Hence, more myeloma cells are in a cell cycle state where they are vulnerable to conventional chemotherapy. We do not know if the addition of daratumumab/isatuximab or carfilzomib to the lymphoma regimens was beneficial or not, but it certainly was tolerable. In both patients, we continued treatment combining traditional lymphoma treatments with myeloma treatment to mitigate the recurrence of the myeloma.

We suggest that EPOCH combined with other less cytotoxic myeloma drugs, such as anti-CD38 antibodies or proteasome inhibitors, is a possible option for anaplastic myeloma, and perhaps also for other rapid growing variants of multiple myeloma, e.g., plasma cell leukaemia. A possible way forward for aggressive myelomas could be to use Dara-EPOCH to bring the patient in remission, followed by bi-specific antibodies such as talquetamab as maintenance therapy.

## Author contribution

ES treated the patients, collected data, and Writing - review & editing. AD treated the patients, collected data and wrote the first draft of the manuscript.

## Data availability

Not applicable.

## Conflicts of interest

Stormorken: No conflicts of interest.

Dahm: Honoraria lecturer of Pfizer, Novartis, Bayer, Bristol Myers Squibb. Honoraria consultant of Pfizer, Bristol Myers Squibb, MSD, Jazz Pharmaceuticals, Abbvie.
